# Cardio-Oncology Educational Program: National Survey as the First Step to Start

**DOI:** 10.3389/fcvm.2021.697240

**Published:** 2021-08-02

**Authors:** Sergey Kozhukhov, Nataliia Dovganych

**Affiliations:** SI “National Scientific Center “The M.D.Strazhesko Institute of Cardiology, ””Kyiv, Ukraine

**Keywords:** cardio-oncolody, cardiotoxicity, educational cardio-oncology program, survey, cancer

## Abstract

**Aim:** The collaboration of cardiologists, general practitioners (GPs), and oncologists is crucial in cancer patient management. We carried out a national-based survey—The Ukrainian National Survey (UkrNatSurv)—on behalf of the Cardio-Oncology (CO) Working Group (WG) of the Ukrainian Society of Cardiology to analyze the level of knowledge in cardio-oncology.

**Methods:** A short questionnaire was presented to specialists involved in the management of cancer patients across the country. The questionnaire was made up of eight questions concerning referred cancer patient number, CV complications of cancer therapy, diagnostic methods to detect cardiotoxicity, and drugs used for its treatment.

**Results:** A total of 426 questionnaires of medical specialists from different regions of Ukraine were collected and analyzed; the majority of respondents were cardiologists (190), followed by GPs (177), 40 oncologists (mainly chemotherapists and hematologists), other −19 (imaging specialists, neurologists, endocrinologists, etc.). All responders were equally involved in the management of cancer patients. However, less than half of the patients have been seen before the start of cancer therapy. GPs observe the majority of patients after the end of treatment. All doctors are sufficiently aware of cancer therapy-associated CV complications. However, the necessary diagnostic tools, mostly biomarkers, are not used widely by different specialists. The criteria for cardiotoxicity, in particular, the level of reduction of the left ventricular ejection fraction (LVEF) as a marker of LV dysfunction, are not clearly understood. The specific knowledge in the management of CV complications in cancer is required.

**Conclusion:** UkrNatSurv is the first survey in Ukraine to investigate the awareness of CO care provided to cancer patients with CV diseases (CVD) or developed CV complications. Providing such surveys among doctors involved in CO is an excellent tool to investigate the knowledge gaps in clinical practice. Therefore, the primary task is to develop a national educational CO program.

## Introduction

Rapidly evolving early detection and novel cancer therapies have significantly reduced mortality. However, survival depends not only on the effective cancer treatment but also on the prevention, diagnosis, and management of complications associated with cancer therapy.

Cancer treatment can affect the CV system in many ways inducing heart failure (HF), arterial hypertension, myocardial ischemia, arrhythmias, thromboembolism, etc. (1).

The development of cancer therapy-associated cardiac complications reduces the quality of life and survival in potentially cured patients, especially in those with a history of CVD.

According to the standards of care, patients with malignancy are managed in cancer centers. However, cancer patients with comorbidities and CV complications during anticancer therapy refer to cardiologists or general practice doctors (GPs).

CV toxicity is a relevant problem among many classes of chemotherapeutic drugs. According to the ESC Position Paper on CV toxicity, nine CV complications of antitumor treatment are classified (1, 2).

However, what the range is of such CV complications in Ukraine, doctors of what specialties manage these patients, what diagnostic methods and drugs do they use in actual clinical practice, and what position statements and guidelines are they acknowledged with?

This is the first survey in Ukraine evaluating the awareness and activity of medical care providers involved in cancer patient management.

It is crucial to identify the level of knowledge of the specialists involved in cardio-oncology to get potential benefit from this service.

It is believed that the study results will figure out vital information to develop an educative CO program and to improve the level of care for cancer patients.

## Methodology

The Ukrainian National Survey (UkrNatSurv) is the study that investigates how to evaluate and manage CV complications in cancer patients in the routine clinical practice setting among doctors of different specialties.

The survey was planned by CO WG of the Ukrainian Society of Cardiology and provided by CO Center of the National scientific center “The M.D.Strazhesko Institute of Cardiology.”

Data were collected through the paper questionnaires provided to the doctors involved in CO across the main country regions during the years 2019–2020. The ethics committee approved the study.

The questionnaire included eight single or multiple-choice structured questions concerning the number of referred cancer patients, CV complications of cancer therapy, diagnostic methods for cardiotoxicity detection, drugs used for cardiotoxicity treatment, etc.

When filling in the answers to the questionnaire, several items were allowed to be selected.

The survey data were entered into a database on the RedCap platform. We used descriptive statistics to summarize these data.

## Results

In total, 426 responses from different regions of Ukraine were collected and analyzed.

**Figure 1:**
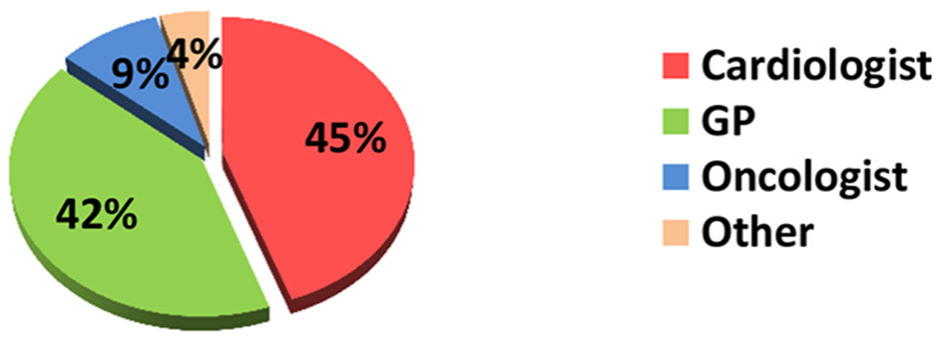
What is your specialty?

**Figure 2:**
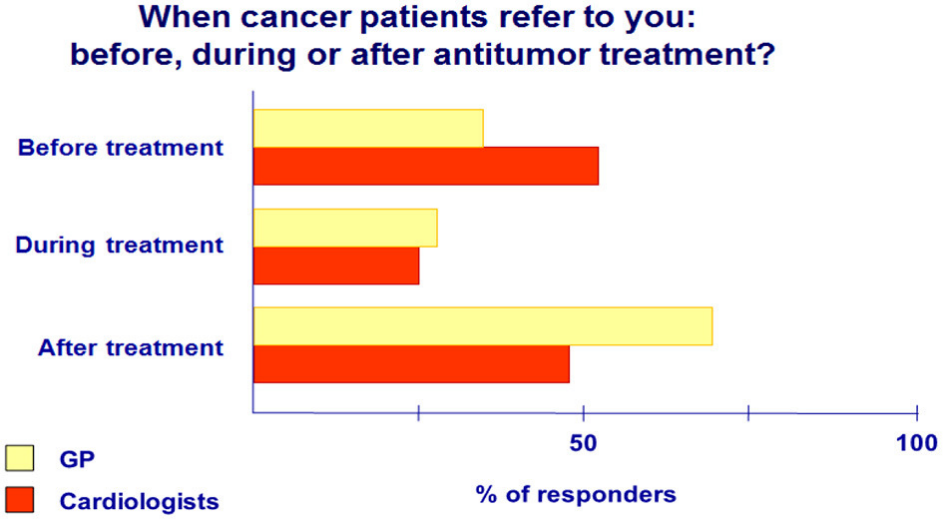
When do cancer patients refer to you: before, during or after antitumor treatment?

**Figure 3:**
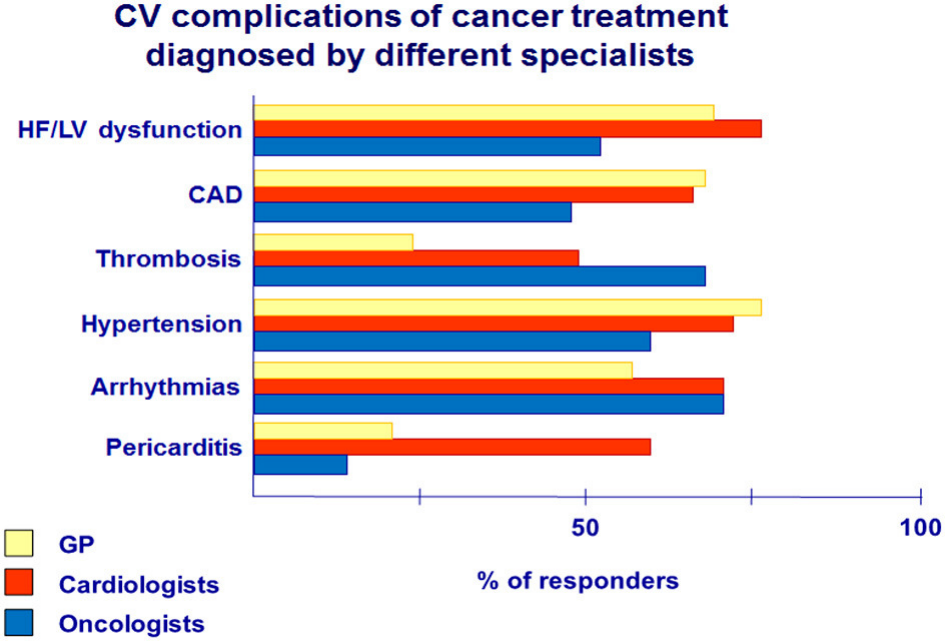
CV complications of cancer treatment diagnosed by different specialists.

**Figure 4:**
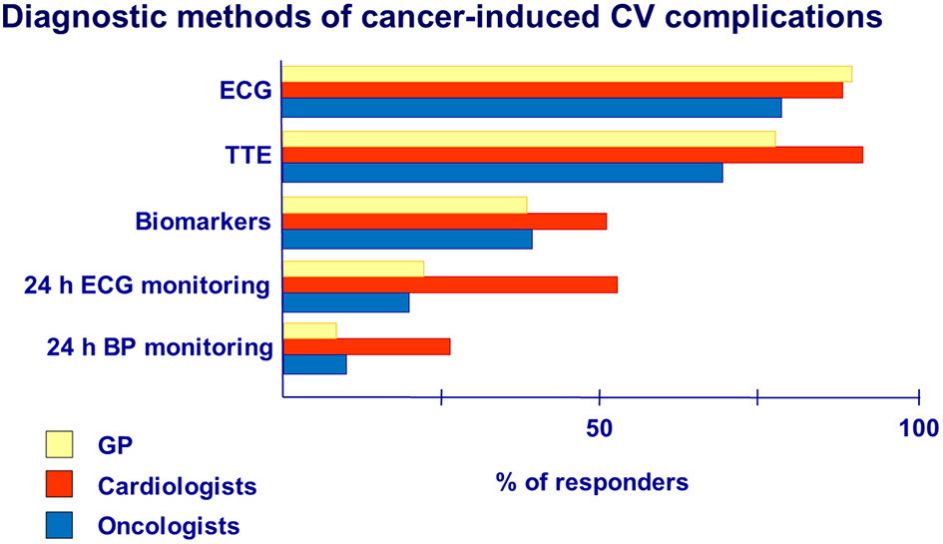
Diagnostic methods for detection of cancer-induced CV complications.

**Figure 5:**
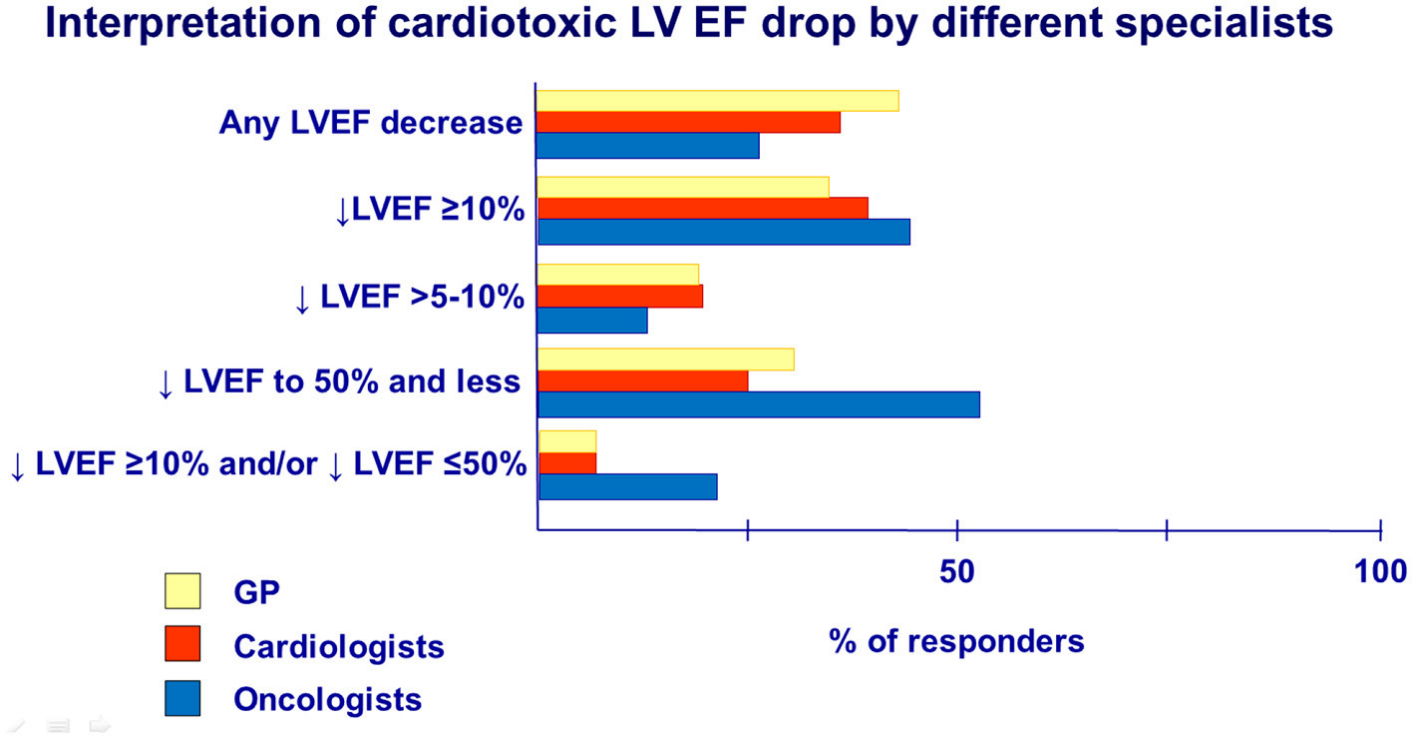
Interpretation of cardiotoxic LV EF drop by different specialists.

**Figure 6:**
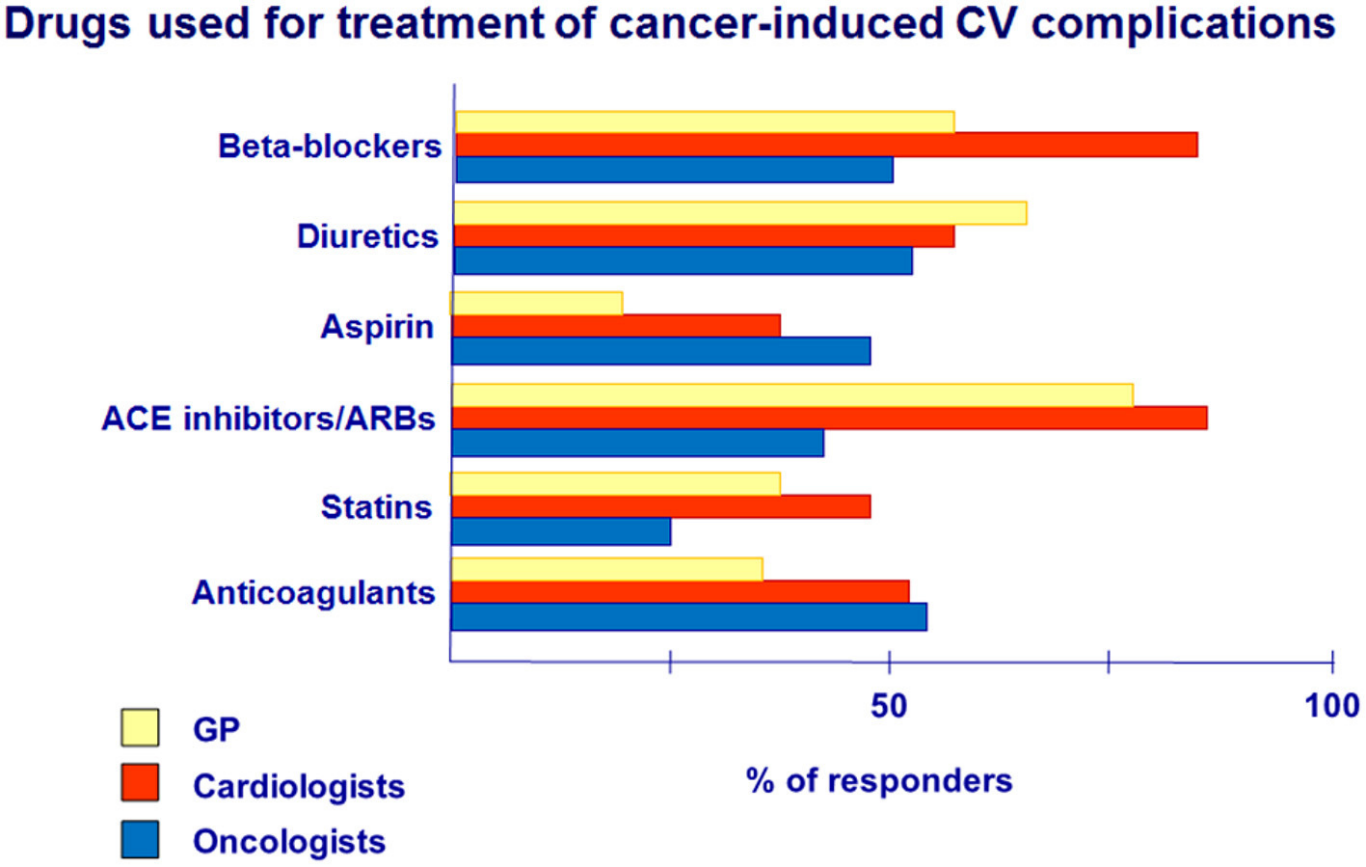
Drugs used for the treatment of cancer-induced CV complications.

**Figure 7:**
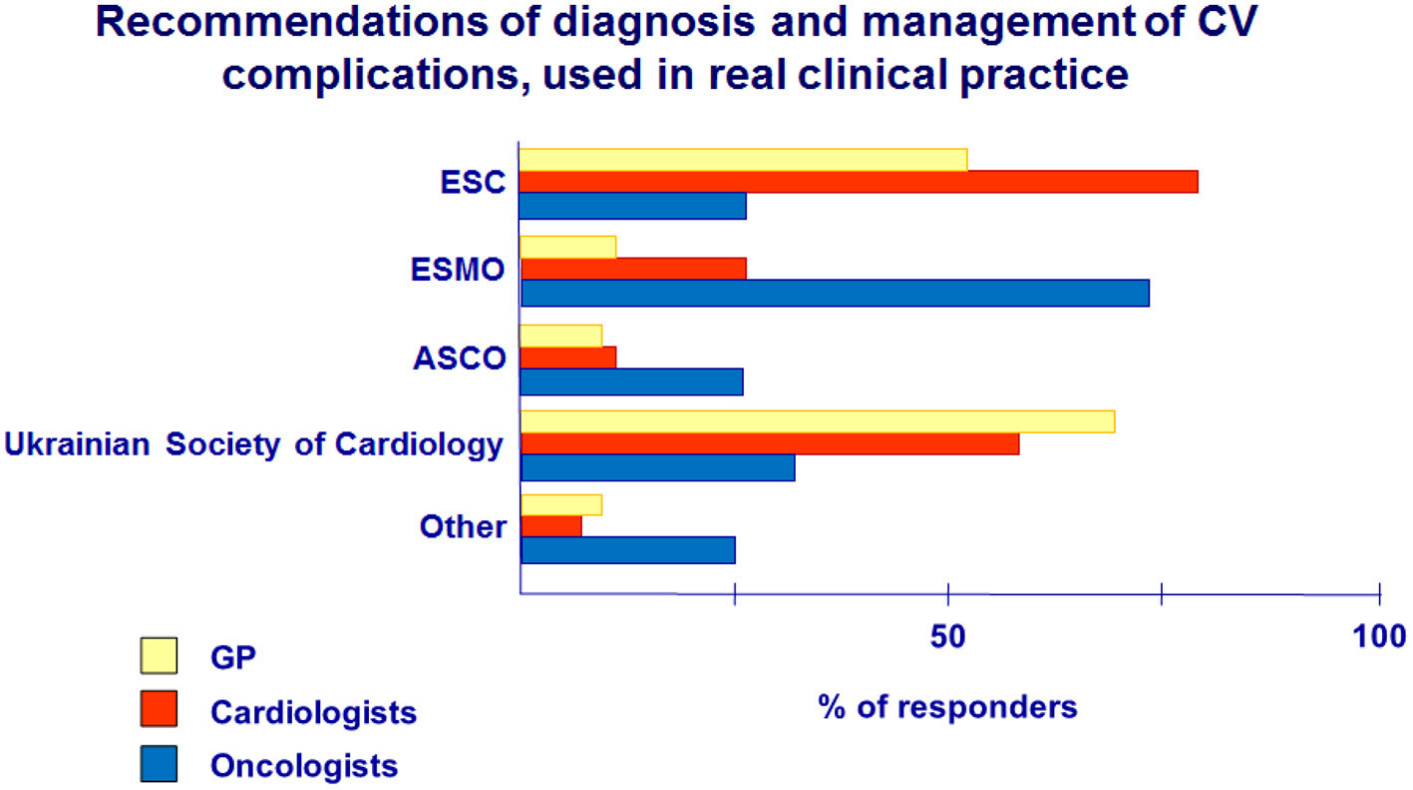
Recommendations for diagnosis and management of CV complications of cancer treatment, used in real clinical practice.

**Question 1. What is your specialty?**
The majority of respondents were cardiologists (*n* = 190, 45%), followed by GPs (*n* = 177, 42%), and 40 (9%) oncologists (mainly chemotherapists and hematologists). The remaining 19 (4%) identified themselves as “others,”—neurologists, imaging specialists, endocrinologists, etc. (Figure 1).
**Question 2. How many patients with a CV complication of cancer treatment have you managed per month?**
Our findings indicate that cardiologists, oncologists, and GPs are equally involved in managing cancer patients. On average, all specialists consult from 5 to 10 patients per month.
**Question 3. When do cancer patients refer to you: before, during, or after antitumor treatment?**
Data analysis revealed that 52% of cancer patients are referred to cardiologists before the start of antitumor treatment; however, they observe only a quarter of these patients during cancer therapy. GPs examine 38% of cancer patients before starting antitumor therapy, less in the cancer treatment process (28%), but manage them predominately (69%) after completion of therapy (Figure 2).
Oncology patients may have CVD or preexisting risk factors that can lead to CV complications mainly due to cancer therapies. The role of a cardiologist or GP in cancer patient management includes prechemotherapy cardiac risk assessment, prevention, identification, and treatment of cardiotoxic complications (3).
**Question 4. What is the main reason for cancer patients' referral: heart failure (HF), coronary artery disease (CAD), VTE, hypertension, arrhythmias, or pericarditis?**
The main CV complications during antitumor therapy are presented in Figure 3.
HF—the most common complication of cancer treatment—is diagnosed mainly by cardiologists compared with GPs (80 vs. 69%) and oncologists-−55%.
Arterial hypertension and CAD in cancer patients had the highest detection rate among GPs (77 and 67%) and cardiologists (71 and 69%) compared with oncologists (60 and 47%). Hypertension is an established risk factor for cardiotoxicity (1, 2). Both cardiologists and GPs need to be informed about careful blood pressure monitoring and more aggressive antihypertensive treatment, especially in patients receiving VEGF inhibitors, due to their effect on blood pressure increase (4).
Severe complication, such as pericarditis, was detected and observed mainly through cardiologists (33%).
Oncologists often face thrombosis (70%) and prescribe anticoagulants for cancer patients, but the majority of those patients are referred then to cardiologists. In addition, both oncologists (70%) and cardiologists (70%) detected arrhythmias more often than GPs (57%).
**Question 5. What diagnostic tools [ECG, transthoracic echocardiography (TTE), 24-h ECG, blood pressure monitoring, and biomarkers] do you provide in patients with cardiac complications during cancer therapy?**
According to the survey data, ECG was the primary method used to diagnose CV complications of cancer therapy in the practice of cardiologists (91%), GPs (93%), and oncologists (83%).
Cardiac imaging, preferably TTE, should be performed at baseline and during therapy in recommended terms depending on the type of anticancer drugs (anthracyclines, trastuzumab, VEGF inhibitors), mainly in patients with preexisting CV diseases and risk factors (1, 5, 6).
Our data showed that TTE in cancer patients was used predominately by cardiologists (96%) than by GPs (79%) and oncologists (73%) (Figure 4).
Our data showed that 47% of cardiologists, 40% of oncologists, and 34% of GPs used biomarkers to detect cardiotoxicity, namely, troponins and natriuretic peptides, in their practice. However, the use of biomarkers needs to be clarified in detail among specialists, as the timing of shifts in these indicators and their detection will depend on many factors related to cancer therapy and the clinical status of the patient (7, 8).
Twenty-four-hour ECG monitoring may be helpful in patients with a history of arrhythmias or in patients in whom drugs with proarrhythmogenic effect (alkylating agents, ibrutinib, and taxanes) are prescribed in chemotherapy regimens. In our study, arrhythmias were presented in the practice of cardiologists (70%), GPs (57%), and oncologists (70%) (Figure 3). However, according to the survey, 24-h ECG monitoring was performed mainly by cardiologists (22%) and not widely.
Although hypertension is one of the well-known complications of cancer therapy, 24-h blood pressure monitoring has rarely been used by all groups of specialists (from 8% of GPs to 21% of cardiologists).
**Question 6. What criteria of cardiotoxicity do you follow in cancer patients with LV dysfunction or HF?**
Recent recommendations of the European Society of Cardiology (ESC) and the European Society of Medical Oncology (ESMO) accept cancer therapy-related cardiac dysfunction as a decline in LV EF of 10% points from baseline to an absolute value of <50% according to repeated evaluations by TTE or cardiac magnetic resonance imaging, as most previous studies were based on this EF value (1, 9). According to the survey, the awareness about the criteria for LV EF decreases because cardiotoxicity in groups of cardiologists, GPs, and oncologists had differed (Figure 5).
Survey data indicated oncologists (and hematologists) (22%) to be more acknowledged in determining cardiotoxic cardiac dysfunction by LV EF and its reduction degree, namely, drop EF >10 percentage points and/or drop EF to ≤ 50%, compared with GPs (6%) and cardiologists (6%). In contrast, the majority of GPs (39%) and cardiologists (33%) selected the answer that any LVEF decrease is a consequence of cardiotoxicity in comparison with oncologists (25%).
**Question 7. What drugs do you usually prescribe to cancer patients with CVD, including those with CV complications?**
Analysis of the use of the drug for CV complication treatment revealed that BB was prescribed significantly more often by cardiologists (85%) compared with GPs (58%) and oncologists (50%) (Figure 6).
At the same time, the use of ACE inhibitors/ARBs among cardiologists and GPs is relatively high and does not differ significantly (85 and 81%, respectively), but they are prescribed twice less by oncologists (40%).
Diuretics for the treatment of CV complications in cancer patients were prescribed by more than 50% of doctors in their practice, mostly by GPs (62%), predominately in patients with HF symptoms.
Our data showed that aspirin had been given more often by oncologists (43%) and cardiologists (37%), while GPs have prescribed aspirin significantly lower (21%). The use of aspirin in cancer patients is recommended, especially in patients with CAD and in patients with multiple myeloma during treatment with lenalidomide/thalidomide (10).
Anticoagulants are the basis of VTE pathogenic treatment (1, 10). According to the survey, oncologists (55%) and cardiologists (52%) have used anticoagulants in cancer patients more often in comparison with GPs (31%).
The issue of statins in cancer patients is controversial. However, data exist about the cardioprotective effect of statins (1).
In our study, cardiologists have prescribed statins more often (46%) compared with GPs (31%) and oncologists (25%). Today, concerning statin therapy in this cohort of patients, it is necessary to follow the general guidelines for managing patients with CV diseases, taking into account risk factors, lipid profile, liver function, etc.
**Question 8. What position statements and guidelines do you follow in routine clinical practice in patients with possible CV complications of cancer treatment?**
Responses to Question 8 indicated cardiologists to be guided by the recommendations of the ESC (78%) and the Ukrainian Society of Cardiology (62%) more often in their practice (Figure 7). GPs mainly used the recommendations of the Ukrainian Society of Cardiology (69%) and ESC (57%). However, there is low awareness of cardiologists and GPs about the recommendations of ESMO and ASCO, but oncologists predominately followed these recommendations (75 and 25%, respectively) in their routine clinical practice.

## Discussion

To date, the world has accumulated extensive experience in the management of cancer patients with CV complications (11, 12). The basis of effective treatment of these patients is a multidisciplinary approach: the team that, along with oncologists (chemotherapists, hematologists, and radiologists), includes cardiologists, GPs, rehabilitation specialists, psychologists, nurses, etc. (13–15).

We conducted a national survey to investigate the awareness in the management of cancer patients with CVD and CV complications among doctors of different specialties in real clinical practice and to understand the gaps in the knowledge.

After the cancer diagnosis establishment, patients should be evaluated for risk factors, CVD, and heart function (1). This will facilitate the detection of CV complications in cancer treatment by comparing the initial data and choosing appropriate monitoring and management for these patients.

During cancer therapy, in case of CV complications, it is necessary to follow a clear algorithm depending on the type of antitumor drug and clinical symptoms because each diagnostic method alone cannot provide complete information about the cardiac status of the patient.

According to the survey, patients are managed mostly by GPs after completion of anticancer treatment, so GPs should be aware of CV complications (HF, VTE) and, if necessary, refer those patients to cardiologists or cardio-oncology centers. Therefore, the GP is an essential member of the multidisciplinary team in the management of cancer patients.

However, follow-up strategies need to be established and adapted for different specialists for better and earlier diagnostic of CV events associated with cancer treatment in a short- or long-term perspective.

Main efforts should be directed on primary prevention strategies to reduce the risk of cardiotoxicity, identification of complications during therapy, and close monitoring after the end of cancer therapy.

LV myocardial dysfunction and HF are the most common complications of antitumor therapy, the clinical manifestations of which may occur during treatment but can develop several years later (1, 2, 5).

In our study, HF was diagnosed by cardiologists (80%), GPs (69%), and oncologists (55%). It is recommended to perform ECG and TTE in cancer patients, predominately with risk factors and CVD, before antineoplastic treatment with potentially cardiotoxic drugs and in a monitoring setting (9).

From this perspective, the determination of LVEF before cancer treatment is crucial because the initial value of the EF will facilitate its drop assessment during cancer therapy and monitoring after treatment completion. It is vital to identify HF/LV dysfunction as early as possible and prescribe cardioprotective therapy for primary prevention or HF treatment.

Survey data indicated oncologists (and hematologists) to be more acknowledged in the determination of cardiotoxic dysfunction by LV EF and its reduction degree. However, data of LV dysfunction knowledge revealed that cardiologists and GPs should be given a more precise definition of LVEF drop criteria because the interpretation of any or slight LVEF decrease as cardiotoxicity may lead to unwarranted patient re-examinations and violation of the timing of cancer treatment, which is essential.

Once the CV complication occurs during antitumor treatment, the patient should consult the cardiologist or GP to prescribe effective cardioprotective therapy and decide on the possibility of further anticancer treatment or changes in the chemotherapy regimens. In our study, prescription of BB and ACE inhibitors by cardiologists and GPs was at high percent. The positive effects of ACE inhibitors and BB were recently evaluated in clinical trials in cancer patients (8, 16–18). It is recommended that ACE inhibitors and BB should be started as early as possible, with appropriate drug dose titration, especially in patients with LV dysfunction due to anthracycline cardiotoxicity (8, 16). As an example, the use of enalapril with carvedilol in the clinical study led to faster LV EF recovery as a response to treatment (17).

VTE occurrence can reach more than 20% in cancer patients. Anticoagulants are the basis of VTE pathogenic treatment (1, 10). Prescription of anticoagulants by GPs was low (31%); therefore, informing physicians about the risks of thrombosis associated with cancer site, the type of antitumor treatment, and personal risk factors is essential in cancer patient management. In addition, the choice of anticoagulant therapy in these patients, its duration, and bleeding control need to be explained more clearly (19).

In recent years, several guidelines and recommendations for clinical practice in cardio-oncology have been issued. Recommendations of the ESC, ESMO, the ASCO, and the European Association of Cardiovascular Imaging (EACVI) are the main documents that justify the decision on detection, monitoring, and treatment of patients during and after cancer therapy (1, 5, 6, 9). In Ukraine, the first National recommendations for managing patients with CV complications during cancer treatment were adapted and published in 2018 at the initiative of the CO Center and the support of the National Cancer Institute. In our study, cardiologists and GPs were guided mainly by the recommendations of the Ukrainian Society of Cardiology and ESC; however, the awareness of ESMO and ASCO recommendations is low, but they are followed mainly by oncologists.

The need for specialists in CO is growing rapidly. Thus, CO requires special knowledge, experience, and dedicated training. In 2020, the CO Leadership Council published a document about education and training in CO that may serve as a roadmap toward CO as a new discipline (20). The authors proposed a three-level CO training.

Based on this approach and the results of UkrNatSurv, we have started the implementation of the first-level CO training program for cardiologists, GPs, and oncologists, which includes basic knowledge on the assessment and management of cancer patients.

However, government support is needed to make this training program available for doctors involved in cardio-oncology across the country.

Additionally, the development of local clinical protocols, recommendations for cancer patient management, and their implementation in real clinical practice should be provided. The Ukrainian CO WG has published recommendations on VTE in cancer, CV complications in breast cancer treatment, and HF in cancer.

Such initiative as providing surveys will give understanding about how to provide optimal care for the cancer patient population.

## Limitations

Survey results and implications of findings are discussed.

The data of this study are not directly representative of the whole country. It was not possible also to assess regional differences.

We suppose that the survey had higher uptake by specialists who were interested and experienced in cardio-oncology. GPs were less likely to participate if they did not have confidence in their knowledge of this field.

These limitations should inform clinicians on the importance of ongoing educational activity and updated guidelines to assist in clinical decision making.

## Conclusion

UkrNatSurv is the first survey in Ukraine to investigate the awareness of cardio-oncology care provided to cancer patients. The study results indicated cardiologists and GPs to be equal players in the cardio-oncology team. However, more clear recommendations for managing cancer patients with CV complications should be published and implemented among these specialists.

Therefore, the priority is to develop a national CO educational program in accordance with the statement of the American College of Cardiology CO Council.

Results of the survey underlined that it is crucial to identify the level of knowledge of the specialists involved in cardio-oncology to get benefit from this service. Different grades of training program will be proposed for the specialists in order to upgrade their experience.

A multidisciplinary approach to cancer patient management, stratification of CV complications before cancer treatment, careful monitoring during treatment, and subsequent long-term monitoring are the key points to improving the survival, quality, and life expectancy of cancer patients.

## Data Availability Statement

The raw data supporting the conclusions of this article will be made available by the authors, without undue reservation.

## Ethics Statement

The studies involving human participants were reviewed and approved by Ethics committee SI National Scientific Center The M.D.Strazhesko Institute of Cardiology. Written informed consent for participation was not required for this study in accordance with the national legislation and the institutional requirements.

## Author Contributions

SK and ND conceptualized and designed the study, collected, analyzed, and interpreted the data. ND drafted the article. SK made critical revisions to the article and approved the final version to be published. All authors contributed to the article and approved the submitted version.

## Conflict of Interest

The authors declare that the research was conducted in the absence of any commercial or financial relationships that could be construed as a potential conflict of interest.

## Publisher's Note

All claims expressed in this article are solely those of the authors and do not necessarily represent those of their affiliated organizations, or those of the publisher, the editors and the reviewers. Any product that may be evaluated in this article, or claim that may be made by its manufacturer, is not guaranteed or endorsed by the publisher.
